# Parathyroid apoplexy, the explanation of spontaneous remission of primary hyperparathyroidism: a case report

**DOI:** 10.1186/1757-1626-2-6399

**Published:** 2009-03-10

**Authors:** Eleni I Efremidou, Michael S Papageorgiou, Evdoxia Pavlidou, Konstantinos J Manolas, Nikolaos Liratzopoulos

**Affiliations:** 1 First Department of Surgery, University General Hospital of Alexandroupolis, Democritus University of Thrace, Alexandroupolis, Greece

## Abstract

Primary hyperparathyroidism due to parathyroid adenoma represents an endocrine disease that is usually treated by surgical intervention (parathyroidectomy). In a very few patients, primary hyperparathyroidism can be spontaneously remit either by infraction or hemorrhage of the adenoma, a fact that is almost certain that will not lead to complete and definite cure. We describe a similar case of a 59-year-old male patient who underwent surgery for a cystic degeneration of a parathyroid adenoma, with substantial preoperative reduction of parathyroid hormone and calcium serum levels, and the diagnostic and treatment modalities are discussed, with a brief review of the current literature.

## Introduction

Primary hyperparathyroidism (PHPT) is a disease that is characterized by elevated calcium and parathyroid hormone (PTH) levels. The disease affects almost all organs and it is presented with a variety of symptoms and signs, ranging from asymptomatic PHPT to a potentially lethal course. The dominant cause of PHPT is the parathyroid adenoma (â‰ˆ85% of cases) and the optimal treatment is the surgical removal of the lesion. However, in the literature there have been a few cases reported, that haemorrhage or infraction in the lesion led to spontaneous remission of hyperparathyroidism. We report a similar case, where a patient was operated on for PHPT, with reduced PTH values postoperatively, compared to the previous ones, and a cystic adenoma with necrotic regions was found and removed.

## Case presentation

**Figure 1:**
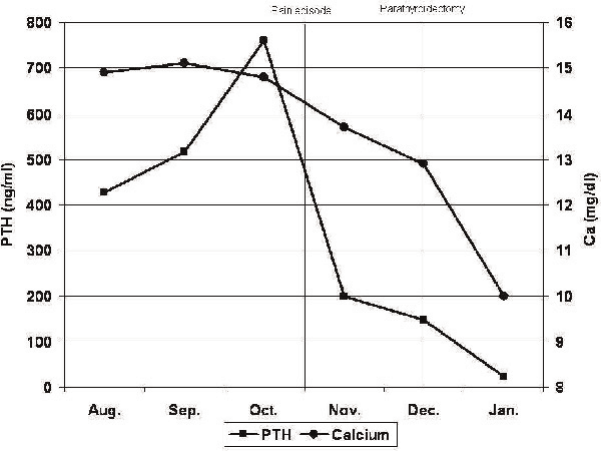
Serum calcium and PTH levels

A 59-year-old Caucasian Greek male presented complaining for arthralgias and peptic ulcer disease for 4 years. From his personal history, he had hypertension and an appendectomy 42 years ago. Clinical examination revealed no abnormal findings from the cervical region, while laboratory examinations revealed elevated calcium (14.9 mg/dl, normal values 8.8-10.6 mg/dl) and PTH levels (426.2 pg/ml, normal values 15-65 pg/ml), thus placing the diagnosis of PHPT, while gastrin level was normal. The patient was advised for further studies for the localisation of the parathyroid pathology and surgical intervention, which the patient refused due to personal reasons. Regular monthly laboratory examinations were conducted for 2 months, which showed elevation of calcium and PTH levels, and 2 months later, the patient consented to further investigation. When the patient presented for his programmed examinations, he reported an episode of sudden cervical pain, without any other symptoms, a few days ago, and the calcium and PTH levels were found to be less elevated than the previous ones (Figure 1). Ultrasonography showed only a nodule measuring â‰ˆ 1 cm of the left lobe of the thyroid gland, scintigraphic examination with sestamibi did not show abnormal uptake, while CT showed the nodule of the thyroid gland and a lesion measuring 22 Ã— 18 Ã— 29 mm in the right cervical region. Surgery was proposed to the patient, at which he did not consent again and he returned for surgery 2 months later. In the preoperative evaluation, the calcium and PTH levels were even more reduced than the previous ones, though they were not in the normal range (calcium level 12.9 mg/dl and PTH level 146.5 pg/ml). A cervical exploration was conducted by an experienced endocrine surgeon (KJM) and his surgical team, in which a cystic lesion measuring 3.5 cm in diameter was removed from the right cervical area, in the anatomic location of the right inferior parathyroid gland, while the rest three glands were identified to be hypoplastic. Moreover, due to pathology of the thyroid gland (multiple nodules), a total thyroidectomy was also performed. Cytological examination of the lesion showed typical parathyroid adenoma cells surrounding a central region of cystic degeneration, thus supporting the diagnosis of a previous parathyroid infarction. The patientâ€™s postoperative course was uncomplicated and he was discharged on the 5^th^ postoperative day, with normal calcium and PTH levels, while in the monthly postoperative follow-up the calcium and PTH levels were found to be normal as well.

## Discussion

The treatment of choice for patients with primary hyperparathyroidism due to parathyroid adenoma is conventional bilateral neck exploration and surgical removal of the lesion. Nowadays, minimally invasive techniques for the management of parathyroid adenomas are gaining ground, although they are not yet widely accepted to become the golden standard for the disease.

In the literature, only 52 cases are reported, in the form of case reports or case series, where spontaneous remission of the PHPT was observed [1], beginning with Norris et al in 1946 [2]. The remission in these cases is usually attributed to hemorrhage or infarction in the adenoma [3]-[8]. Other authors [9] propose that the hemorrhage and infarction are two stages of the same phenomenon, which begins with necrosis and variable hemorrhage, the extent of which defines the clinical course. This phenomenon is referred to as â€œautoparathyroidectomyâ€� or â€œparathyroid apoplexyâ€� due to similarities with pituitary apoplexy [10] and Nylen et al [9] proposed a classification of parathyroid apoplexy, according to the extent of the hemorrhage and the clinical features (Table 1). Elangovan et al [11] also proposed that rapid growth rate of the adenoma can lead to a vascular event insufficient to produce infarction, but sufficient to reduce oxygen supply and consequently to a reduction in its capacity to produce excessive parathyroid hormone, based on case reports that the pathology report did not show any regions of necrosis or evidence of hemorrhage.

**Table 1:** Classification of parathyroid apoplexy proposed by Nylen et al [9]

Type	Pathogenesis	Clinical features	Outcome
**I**	Adenoma with necrosis without hemorrhage	Asymptomatic, GI discomfort/pain, joint pain, cervical pain	Initial hypercalcemia- hypocalcemia
II	Adenoma with intracapsular hemorrhage and necrosis	Asymptomatic, GI discomfort, cervical pain, tetany, convulsions	Initial hyper- or hypocalcemia
III	Adenoma with extracapsular hemorrhage	Acute pain (neck to substernal), dysphagia, dysphonia, hoarseness, stridor, hypercalcemic crisis, widened mediastinum, visible hematoma or ecchymoses	Stable postoperative course, normocalcemia

Parathyroid apoplexy can lead to spontaneous remission of hyperparathyroidism, although in many cases the course of the phenomenon can be milder and can result only in asymptomatic reduction of calcium and PTH levels [12]. Most of the reported cases presented with acute symptoms of hypocalcemia with or without tetania, resulting from the adenomaâ€™s necrosis and the incompetence of the remaining parathyroid glands to produce the necessary amount of PTH or hypercalcemia due to excessive PTH release, followed by a period of normocalcemia at first and finally recurrence of the disease. In rare cases, patients can present with signs and symptoms of massive cervical or mediastinal hemorrhage due to extracapsular hemorrhage of the adenoma, a condition that requires emergency neck exploration surgery. In the majority of the cases reported in the literature, surgical treatment was ultimately performed, while only a few patients were treated conservatively, under periodical clinical and biochemical follow-up [7,8].

Based on the data, in cases of PHPT with spontaneous reduction of PTH and calcium serum levels, the hypothesis of â€œparathyroid apoplexyâ€� can be supported only by a histopathological report of the adenomaâ€™s necrosis and degeneration.

In our case, the episode of cervical pain and the automatic reduction of calcium and PTH levels, although they did not reach normal values, seem to support the hypothesis of incomplete necrosis of the parathyroid adenoma. This suggestion is also supported by the fact that Sestamibi scan after the episode did not show any clear evidence of an adenoma, while the histopathology report of the surgical specimen documented the cystic degeneration of the adenoma.

Authorsâ€™ suggestion, which comes in agreement with the previously reported data, is to perform parathyroidectomy rather than conservative treatment in all cases of suspected â€œparathyroid apoplexyâ€�, at any stage of the disease, in order to have a definite diagnosis and permanent solution of PHPT. Moreover, authorsâ€™ belief is that the preferred surgical approach should be conventional bilateral neck exploration by a specialist surgeon in the endocrine surgery field, rather than minimal invasive surgery, an approach which offers the advantage of identification and examination of the remaining parathyroid glands.

## Consent

Written informed consent was obtained from the patient for publication of this case report. A copy of the written consent is available for review by the Editor-in-Chief of this journal.

## Competing interests

The author(s) declare that they have no competing interests.

## Authorsâ€™ contributions

EIE conceived the case report, analyzed and interpreted the patient data. MSP collected the data and was a major contribution in writing the manuscript. EP participated in drafting the manuscript. KJM operated on the patient, reviewed the manuscript, and gave final approval. NL participated in the operation, conceived the case report, and drafted the manuscript. All authors read and approved the final manuscript.
